# Evaluation of heavy metal concentration in imported black tea in Iran and consumer risk assessments

**DOI:** 10.1002/fsn3.1267

**Published:** 2019-11-17

**Authors:** Fatemeh Pourramezani, Fateme Akrami Mohajeri, Mohammad Hossein Salmani, Arefe Dehghani Tafti, Elham Khalili Sadrabad

**Affiliations:** ^1^ Zoonotic Diseases Research Center Department of Food Hygiene and Safety School of Public Health Shahid Sadoughi University of Medical Sciences Yazd Iran; ^2^ Department of Environmental Health Engineering School of Public Health Shahid Sadoughi University of Medical Sciences Yazd Iran; ^3^ Department of Biostatistics School of Public Health Shahid Sadoughi University of Medical Sciences Yazd Iran

**Keywords:** heavy metals, Indian tea, Iran, Sri Lankan tea

## Abstract

Tea grows in the contaminated soils, absorbs the heavy metals, and enters them into the human food chain. The concentrations of Pb, Cd, Cu, As, and Hg of the imported black tea leaves to Hormozgan Province were evaluated by atomic absorption spectrometer. Then, the Hazard Quotient (HQ) and Hazard Index (HI) levels of heavy metal intakes were calculated to estimate the health hazard for consumers. The Pb, Cd, Cu, As, and Hg concentrations in the Sri Lankan and Indian blank tea were 0.14, 0.017, 11.29, 0.057, 0.0076 mg/kg, and 0.21, 0.02, 14.56, 0.067, 0.01 mg/kg, respectively. It was found that except for As concentration in Indian black tea were higher than Sri Lankan black tea. The HQ and HI levels of all studied metals were less than one, but they were higher in Indian black tea compared with the Sri Lankan black tea. The HI of Indian and Sri Lankan black tea samples were 0.061 and 0.048, respectively, which indicated no significant health hazard for tea consumers. The results showed that the consumption of the studied tea could not have any risk of heavy metal exposure.

## Graphical abstract

The Pb, Cd, Cu, As, and Hg in both Sri Lankan and Indian were lower than standard limit. The HQ and HI levels of all studied metals were less than one in Iranian consumers. The heavy metals of imported black tea to Iran were investigated. According to our results, there was no problem in terms of heavy metal contamination.

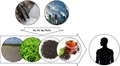

## 1 INTRODUCTION

Tea is produced by the leaves of three types of *Camellia sinensis*, *Camellia assamica*, and *Cambodiensis* from the family of *Theeaceae* (Mehra & Baker, 2007). The tea has been used in traditional medicine for centuries due to its pharmacological properties and beneficial for human health (Das, de Oliveira, da Silva, Liu, & Ma, 2017; Mehra & Baker, 2007). Over the last decades, global production of this plant has a growth rate of higher than 1.8% and global tea consumption has increased more than two percent per year (Karimzadeh et al., 2013). Tea is considered as the second popular beverage in the world because of its taste and health properties (Das et al., 2017; Qin & Chen, 2007). Iran is one of the most important global producers and consumers of black tea which consume about five percent of the world's tea; each Iranian drinks about 1.5 kg of black tea per person per year (Salahinejad & Aflaki, 2010; Yousefi, Jahangard, & Mahmoudian, 2017).

Ingestion of food and beverage is considered as the important pathway of human contamination by heavy metals (Das et al., 2017). Therefore, the presence of heavy metal in tea is one of the most important indicators to evaluate the tea quality (Zhang et al., 2018). The tea contamination could occur during growth in the polluted soil and manufacturing processes (Karimi et al., 2008). Moreover, human activities such as mine exploitation, melting, and other industrial and agricultural activities increase the concentration of heavy metals by releasing them in the environment (Nkansah, Opoku, & Ackumey, 2016. By brewing the tea, heavy metals can be transferred into tea infusion and regular consumption of tea can enter them into the human body which could cause adverse effects on human health (Nkansah et al., 2016; Zhang et al., 2018). World Health Organization introduced arsenic (As), lead (Pb), mercury (Hg), and cadmium (Cd) among 10 chemicals which considered as major public health concerns (RoyChowdhury, Datta, & Sarkar, 2018). The presence of excess amounts of heavy metals such as Pb, As, Cu, Cd, and Hg in tea can increase the concentration of toxic metals in the human body. The excessive intake of these elements by humans leads to poisoning and causes various complications, such a renal failure, methemoglobinemia, liver cirrhosis, and impairment in the formation of red blood cells (Escott‐Stump, Mahan, & Raymond, 2012).

It has been estimated that about 50 percent of black tea in Iran market were imported from different countries. Regarding the habit of drinking tea among Iranian, evaluation of the heavy metal concentration and their maximum allowable levels in tea as a basic part of health risk assessment could be effective on the protection of human health (Cao, Qiao, Zhang, & Chen, 2010). So, the objective of the present study was the evaluation of heavy metal (Pb, Cd, Cu, As, and Hg) concentration in black tea leaves imported to Iran from India and Sri Lankan at 2017 by atomic absorption spectrometry. Consequently, the health risk of these heavy metals in Iranian tea consumers was assessed by Hazard Quotient (HQ) and Hazard Index (HI) methods, which show the potential noncarcinogenic effects of individual and multiple metals, respectively (Zhang et al., 2018).

## 2 MATERIAL AND METHODS

### 2.1 Apparatus

All glasswares were washed and rinsed by distilled water to remove any particles. Then, they were soaked and kept overnight in 10% (v/v) nitric acid (Merck). After that, the glasswares were washed with distilled water, rinsed, and dried before use (Sadrabad, Boroujeni, & Heydari, 2018). The atomic absorption spectrometer (SavantAA) was used to determine the concentration of heavy metals. The operational characteristics for measurement of each heavy metal are mentioned in Table 1.

**Table 1:** Operating parameters for the atomic absorption spectrophotometer

Parameter	Pb	Cd	Hg	As	Cu
Wavelength (nm)	217	228.8	253.7	193.7	324.8
Silt (nm)	1	0.5	0.5	1	0.5
Lamp current (mA)	5	3	4	8	3

### 2.2 Sampling

The sampling was done according to Institute of Standards and Industrial Research of Iran (ISIRI, 2008). A total of 122 black tea samples were randomly collected from local market of Hormozgan, Iran. The origin of black tea samples was from India and Sri Lanka.

### 2.3 Sample digestion and analysis

For determination of Pb, Cu, and Cd, the dry digestion was done by burning 5 g of each tea powder sample by an electric furnace at 450 ± 5°C for 8 hr. The residuals were solved in 5 ml 37% (v/v) hydrochloric acid (Merck) and diluted by deionized distilled water to reach 50 ml volume. The Pb and Cd contents of samples were analyzed using graphite furnace atomic absorption spectrometer (GFAAS, SavantAA GBC). The Cu content of samples was evaluated by flame atomic absorption spectrophotometer (ISIRI, 2007).

For As determination, 5 g of each sample was mixed with MgNO_3_ and 6H_2_O 8MgO and 32% nitric acid (Merck) to a ratio of 20:5, then, the mixture was put on hotplate (100 ± 5°C) until it was evaporated. Then, the mixture was put into electric furnace (425 ± 25°C) for 12 hr. The heating by electric furnace (400°C) was continued for 2 to 3 hr along with adding 5 ml nitric acid (65%). The dried samples were treated by distilled water and mixture of potassium iodide and ascorbic acid in HCl (6M), in a ratio of 1:5:5 ml. The obtained solution was filtered through 0.45 µm membrane filter and diluted to 25 ml using HCl (6M). The As content of samples were measured by hydride generation atomic absorption spectrophotometer (ISIRI, 2013).

In order to measure Hg, 5g of the powdered samples were weighted. Then, nitric acid (7M), sulfuric acid (9M), and sodium molybdate (2%) in a proportion of 20:25:1 ml were added to each sample. The mixture was heated by electrical heater for 1 hr. The heating was continued by adding 10 ml of distilled water. Then, the digested samples were filtered and reached 100 ml by sulfuric acid solution (0.5M). The cold vapor atomic absorption spectrophotometer was used for the determination of Hg of samples.

### 2.4 Health hazards estimation

The Hazard Quotient (HQ) of each metal was evaluated for the determination of noncarcinogenic health risks in Iranian tea consumer. The HQ less than 1 shows no significant risk of noncarcinogenic effects in consumers (Zhang et al., 2018). The HQ is calculated by equation described by Cao et al as follows (Equations 1 and 2) (Cao et al., 2010).

(1) HQ=ADD/RfD

(2) ADD=C×IR/BW

where ADD is the average daily intake of metal (μg/kg/day) and RFD is the daily intake reference dose. According to Environmental Protection Agency (EPA), RfDs for Cd, Cu, and As are 1, 40, and 0.3 μg kg^−1^ day^−1^, respectively (Li, Fu, Achal, & Liu, 2015). The rate of RfD was not calculated by US‐EPA for Hg and Pb. In this study, the provisional tolerable weekly intake (PTWI) given by WHO was used for Hg (5 μg kg^−1^ week^−1^) and Pb (25 μg kg^−1^ week^−1^), respectively (Cao et al., 2010).

In above Equation 2, *C* is the mean concentration of metals and IR is the consumption rate which was considered as 6 g/day according to the study was done by (Naghipour, Amouei, Dadashi, & Zazouli, 2016). Furthermore, BW is the average weight of consumers, which was estimated 65 kg.

The Hazard Index (HI) could calculate the overall or interactive effects of exposure to two or more contaminants (Cao et al., 2010; Zhang et al., 2018). Therefore, total noncarcinogenic health risk of multiple contaminants is estimated as bellow (Equation 3) (Cao et al., 2010).

(3) $$\text{HI}={\text{HQ}}_{1}+{\text{HQ}}_{2}+{\text{HQ}}_{3}+\ldots +{\text{HQ}}_{n}$$

### 2.5 Data analysis

The data were analyzed by the SPSS16 software. Mann–Whitney test was used to determine the abnormal information, and the one‐sample *t* test was applied to compare the quantitative values.

## 3 RESULTS

### 3.1 Heavy metal concentration

According to Table 2, Pb, Cd, Cu, As, and Hg were 0.14, 0.017, 11.29, 0.057, and 0.0076 (μg/g) in Sri Lankan tea, respectively, which were less than the acceptable limit. The results (Table 2) show that the level of Pb, Cd, Cu, As, and Hg was 0.21, 0.02, 14.56, 0.067, and 0.01 (μg/g) in Indian tea, respectively. It was indicated that the total amount of metals in Indian tea samples was lower than the acceptable limit. The results of the statistical analysis indicated that Pb, Cd, Cu, and Hg were significantly higher in Indian tea than the Sri Lankan tea samples. However, no significant difference was observed between the Sri Lankan and Indian tea with regard to As.

**Table 2:** Average heavy metals of lead, cadmium, copper, arsenic, and mercury (μg/g) in Indian and Sri Lankan tea samples

Metal	Country	Mean	Minimum	Maximum
Pb	India	0.21 ± 0.169^a^	0.001	0.73
	Sri Lanka	0.14 ± 0.109^b^	0.001	0.6
Cd	India	0.02 ± 0.013^a^	0.0002	0.052
	Sri Lanka	0.017 ± 0.019^b^	0.0002	0.084
Cu	India	14.56 ± 6.85^a^	0.033	52.26
	Sri Lanka	11.29 ± 5.906^b^	0.033	64.24
As	India	0.067 ± 0.036^a^	0.007	0.14
	Sri Lanka	0.057 ± 0.0323^a^	0.0083	0.13
Hg	India	0.01 ± 0.0049^a^	0.0016	0.019
	Sri Lanka	0.0076 ± 0.0046^b^	0.0001	0.019

Note

Different letters for each heavy metal show significant differences at level of *p* < .05.

Limit of Detection (LOD): Hg = 0.000061, Pb = 0.0013, Cd = 0.00021, As = 0.00021, Cu = 0.033.

According to Table 3, the correlation coefficients among the concentrations of metals in black tea samples are given. The metal‐to‐metal correlation was not significant for all samples, and each metal was independent of the other elements, except Cu, Pb, and Cd. In other words, Cu had positive correlation with Pb and Cd.

**Table 3:** Correlation coefficients between metal concentrations (α = 0.05)

	As	Hg	Pb	Cd	Cu
As	1	0.180	0.121	0.170	0.061
Hg		1	0.084	0.002	0.1
Pb			1	0.171	0.370**
Cd				1	0.245**
Cu					1

** Showed significant differences at level of 95%.

### 3.2 Estimation of HQ and HI

The result of Hazard Quotient and Hazard Index are presented in Table 4. The results show that all HQ levels of metals were less than one. Furthermore, we found that the HQ levels of all metals were higher in Indian tea compared with the Sri Lankan tea. The HI values of these metals in Indian and Sri Lankan tea were 0.061 and 0.048, respectively, which indicated no significant noncarcinogenic health hazard in the consumption of black tea for Iranian consumers.

**Table 4:** Estimated Hazard Quotient and Hazard Index of exposure to metals

Country	Parameters	Pb	Cd	Hg	Cu	As
India	ADD	1.9 × 10^–3^	1.8 × 10^–3^	9.2 × 10^–4^	1.34	6.1 × 10^–3^
	HQ	5.3 × 10^–3^	1.9 × 10^–3^	1.2 × 10^–3^	3.36 × 10^–2^	2 × 10^–2^
	HI ꞊ ∑ HQ			6.1 × 10^–2^		
Sri Lanka	ADD	1.2 × 10^–2^	1.5 × 10^–3^	7 × 10^–4^	1.04	5.2 × 10^–3^
	HQ	3.5 × 10^–3^	1.5 × 10^–3^	9.8 × 10^–4^	2.6 × 10^–2^	1.7 × 10^–2^
	HI ꞊ ∑ HQ			4.8 × 10^–2^		

## 4 DISCUSSION

Previous studies indicated that the excessive levels of heavy metals in tea and their absorption in the human body can cause poisoning and resulted in several problems (Mahan, Escott Stump, & Raymond, 2012). Due to the role of heavy metal on human health, the concentration of heavy metals such as Pb, Cu, Hg, As, and Cd was evaluated in two samples of the imported tea in Hormozgan Province. Then, the noncarcinogenic effects of individual and multiple metals were determined by the HQ and HI, respectively. The mean concentration of heavy metals in Indian and Sri Lankan black tea showed the following order: Cu > Pb > As > Cd > Hg. Peng et al. showed the mean of Cu in tea was higher than those of Pb and Cd, which is consistent with the results of the present study (Peng et al., 2018). The results showed that the concentration of Pb, Cd, Cu, and Hg in Indian tea samples were significantly higher than those in Sri Lankan tea. However, no significant difference was observed between the Sri Lankan tea and Indian tea in terms of As.

According to Table 2, Pb, Cd, Cu, As, and Hg mean concentrations of Indian and Sri Lankan black tea samples were 0.21 and 0.14, 0.02 and 0.017, 14.56 and 11.29, 0.067 and 0.057, and 0.01 and 0.0076 µg/g, respectively. These levels were less than the standard limits recommended by WHO (Pb: 10 mg/kg, Cd: 3 mg/kg) and Iran national standard organization (Cu: 50 mg/kg, As: 0.15 mg/kg, Hg: 0.02 mg/kg) (ISIRI, 2007; Nkansah et al., 2016; Soliman, 2016).

Qin and Chen reported that the mean concentration of Pb in tea samples of the Chinese market was 1.32 mg/kg, which was higher than the allowable limit of the Chinese Ministry of Health (0.3 µg/g) in some samples (Qin & Chen, 2007). Also, Karimzadeh et al. evaluated the amount of some heavy metals in 32 samples of black tea produced in Mazandaran factories in spring and summer. They found that the concentrations of Pb, Cd, and Cu in spring and summer were 2.33–2.25, 0.4–0.16, and 23.14–30.19 mg/kg, respectively (Karimzadeh et al., 2013). A comparison between the results of these two studies and the current study indicates that the concentration of Cu in Sri Lankan tea (11.29 mg/kg) and Indian black tea (14.56 mg/kg) was lower than Cu concentration in tea plants cultivated in North of Iran. Contrary to the findings of the present study, the Cu concentrations were significantly higher than Iran standards (Karimzadeh et al., 2013). Nasri reported that the concentrations of Pb, Cd, Cu, and As in different tea samples were all in the range of 0.049–10.12, 0.016–0.123, 3.05–37.41, and 0.0431–0.287 mg/kg, respectively. The maximum concentration of Cu in the current study was higher than the results of Nasri et al (Nasri, Amini, & Mohammadi, 2017). The study by Ansari et al. showed that the average Cu in cultivated black tea was 29.3 mg/kg (Ansari, Norbaksh, & Daneshmand, 2007). Naithani and Kakkar (2005) indicated that the concentration of Cu in tea was 11.1 µg/g, which is less than the concentrations in Sri Lankan and Indian tea samples of the current study (Naithani & Kakkar, 2005). Studies conducted in Japan, Turkey, and Saudi Arabia on heavy metals in tea represented that the concentrations of Cu were 27.7, 24.8, and 18.1 mg/kg, respectively, which are higher than the Cu concentration of the current study (Ashraf & Mian, 2008; Matsuura, Hokura, Katsuki, Itoh, & Haraguchi, 2001; Narin, Colak, Turkoglu, Soylak, & Dogan, 2004). Naghipour et al. (2016) evaluated the concentration of heavy metals in different types of black and herbal tea cultivated in North of Iran and found that the concentration of As in black and white tea was in the range of 0.03–0.1 and 0.01–0.03 mg/kg, respectively. These levels are lower than the allowable limits. Furthermore, Shi et al. reported that the concentration of As was in the range of 0.02–0.07 mg/kg in the leaves of green and fresh tea (Shi, Jin, & Zhu, 2007). In another study by Cao et al. (2010), the concentration of As was reported in the range of 0.02–0.07 mg/kg. The concentration of As in the current study is in agreement with the concentrations reported by mentioned studies. Moreover, Santos, Duboc, Goncalves, and Jacob (2015) indicated that the concentrations of As in Brazilian tea leaves and its infusion were in the ranges of 0.4–0.02 and 0.005–0.007 mg/kg, respectively. Karimi et al. (2008) evaluated the concentrations of heavy metals in the tea samples of Iranian markets and reported the average amount of Hg as 0.61 mg/kg, which is not consistent with the results of our study. Barone, Giacominelli‐Stuffler, and Storelli (2016) also studied heavy metals such as Hg, Cd, Pb, Cu, Zn, Ni, Fe, Cr, and Se in various imported tea samples in Italy and showed that the concentrations of metals varied in different brands. They reported that the highest amount of Hg was 0.27 mg/kg in Chinese tea.

These differences in results are due to the difference in cultivation region, soil conditions, height of the land from sea, rainfall, and other environmental conditions (Barone et al., 2016; Karimzadeh et al., 2013). However, the use of phosphate fertilizers and fungicides in agriculture may also contaminate herbal foods with heavy metals. These materials often contain significant amounts of toxic elements such as Hg, Cd, Pb, and Cu (Ferreira et al., 2015; Karak & Bhagat, 2010; Soliman, 2016). The concentration of heavy metals in tea plant is greatly caused by the presence of factors such as combination of metals in soil, the plant species, the pH of the growth environment, and the genetic characteristics of the tea plant (Foy, Chaney, & White, 1978). Although, high amounts of heavy metals in tea can be attributed to dust particles during the production process as well as contaminations during the packaging process (Ashraf & Mian, 2008).

The HQ level of metals in Indian and Sri Lankan black tea showed the order of As > Cu > Pb > Cd > Hg and Cu > As > Pb > Cd > Hg, respectively. The HQ values related to all studied metals were less than one, which shows no hazards to the consumers. The comparison between Indian and Sri Lankan black tea samples with regard to HQ values showed that HQ was higher in all metals of Indian tea sample. In the study of Li et al. (2015) on the health hazards of metals in green tea in China, the HQ levels were similar to those reported in the present study (Al > Cu > Ni > Pb > Cr > Co). Similar results were also indicated by Cao in tea of Puerh city (Cao et al., 2010). As mentioned previously, HI values of less than one indicated that the sample had no health risk. The values of HI in Indian and Sri Lankan tea samples of the present study were 0.061 and 0.048, respectively. In the study by Cao et al. (2010), the amounts of HI in Puerh tea were 0.17 and 0.29 among the consumers of Kunming and Puerh cities, respectively, which are higher than the amounts of HI in the present study. In another study by Zhang et al. (2018) on fresh and mature tea leaves, the amount of HI in fresh leaves was less than one which was consistent with the present study. The HI of five metals of the present study in Indian and Sri Lankan black tea samples indicated no significant health hazards for Iranian consumers.

## 5 CONCLUSION

The findings of the current study indicated that heavy metal concentrations in tea samples were at allowable level. The amounts of HQ and HI were less than one in all studied metals. However, comparison of the HQ values between the Indian and Sri Lankan tea samples showed that the Indian tea had higher HQ values. However, it was showed that the consumption of the Indian and Sri Lankan tea samples is not harmful with regard to the presence of Cd, Pb, Cu, Hg, and As. Considering that Hormozgan Province is placed in a special location and the major imports of the country, it is recommended that imported tea would be controlled, traced, and checked to ensure the health of consumers and provide their psychological and economic security. The active, real, continuous, and reliable monitoring of the administrative organizations will ensure the safety of consumers.

## CONFLICTS OF INTEREST

The authors declare no conflict of interest.

## ETHICAL APPROVAL

The current study does not involve any human or animal testing.
